# Serum RNASE1 as a biomarker for disease activity and lupus nephritis in systemic lupus erythematosus

**DOI:** 10.3389/fimmu.2026.1790455

**Published:** 2026-04-01

**Authors:** Jianping Xiao, Hui Li, Ju Wang, Ruifeng Wang, Fuquan Jin, Deguang Wang

**Affiliations:** 1 Department of Nephrology, The Second Affiliated Hospital of Anhui Medical University, Hefei, Anhui, China; 2 Key Laboratory of Multi-Cell Systems, Center for Excellence in Molecular Cell Science, Shanghai Institute of Biochemistry and Cell Biology, Chinese Academy of Sciences, University of Chinese Academy of Sciences, Shanghai, China

**Keywords:** biomarker, lupus nephritis, RAC score, RNASE1, systemic lupus erythematosus

## Abstract

**Introduction:**

Systemic lupus erythematosus (SLE) is a heterogeneous autoimmune disease characterized by autoantibody production and immune complex-mediated organ damage, frequently involving the kidneys as lupus nephritis (LN). However, reliable blood-based biomarkers for disease activity and renal involvement remain limited.

**Methods:**

We performed serum proteomic profiling in newly diagnosed SLE patients and integrated these data with renal transcriptomic datasets from SLE mouse models to identify candidate biomarkers. Associations between serum RNASE1 levels and clinical parameters were evaluated, and a composite RNASE1-albumin-calcium (RAC) score was developed.

**Results:**

RNASE1 was consistently upregulated in both serum and renal tissues of SLE patients. Serum RNASE1 levels were significantly elevated compared with healthy controls and showed positive correlations with proteinuria and SLE Disease Activity Index (SLEDAI) scores, and a negative correlation with complement C3. The RAC score demonstrated stronger correlations with disease activity and renal function than RNASE1 alone. Receiver operating characteristic (ROC) analysis showed that the RAC score discriminated SLE disease activity with area under the curve (AUC) values of 0.7654, 0.7737, and 0.8072 for ≥mild, ≥moderate, and ≥severe activity, respectively. In addition, renal RNASE1 expression increased with LN severity and was significantly higher in class IV ± V LN than in class III ± V or class V LN, correlating with the pathological activity index.

**Discussion:**

RNASE1 is a promising biomarker reflecting both systemic disease activity and renal involvement in SLE. The RNASE1-based RAC score provides a noninvasive tool for improved disease assessment and may facilitate clinical monitoring and management of SLE patients.

## Introduction

Systemic lupus erythematosus (SLE) is a chronic autoimmune disease characterized by autoantibody production, immune complex deposition, and dysregulated immune responses, leading to multisystem involvement (1, 2). The disease can affect multiple organs, including the skin, joints, nervous system, and kidneys (3, 4). Among these, renal involvement represents one of the most frequent and severe complications, with lupus nephritis (LN) developing in a substantial proportion of patients during the disease course (5, 6). LN is a major determinant of morbidity and mortality in SLE, and accurate assessment of disease activity is essential for guiding treatment and predicting renal outcomes (7).

Renal biopsy remains the gold standard for diagnosing LN and evaluating histological activity, providing critical information on glomerular, tubular, and interstitial lesions that guides treatment decisions and prognostication (8, 9). However, its invasive nature limits repeated assessment and longitudinal monitoring. In clinical practice, serological markers such as complement components (C3 and C4), anti-double-stranded DNA (dsDNA) antibodies, and routine laboratory parameters including proteinuria and serum creatinine are commonly used to evaluate disease activity (8). Nevertheless, these markers have limited sensitivity and specificity, and their fluctuations often fail to fully reflect parallel histological changes in the kidney (10). Therefore, the identification of reliable and noninvasive biomarkers that better capture both systemic immune activation and renal involvement remain an important unmet need in SLE management.

Ribonuclease 1 (RNASE1) is a secreted endoribonuclease predominantly produced by endothelial cells and plays an essential role in the degradation of extracellular RNA (11). Recent studies suggest that extracellular RNA can act as a damage-associated molecular pattern that promotes endothelial activation, leukocyte recruitment, and inflammatory responses (12). By regulating extracellular RNA levels, RNASE1 contributes to vascular homeostasis and modulates immune and inflammatory responses (13). Previous studies have shown that RNASE1 can influence immune cell activation and infiltration, including Th1/Th17 cells and natural killer cells, and may interact with intracellular signaling pathways such as STAT1 to regulate immune responses (14–17). Given that endothelial dysfunction, immune cell infiltration, and inflammatory signaling are central mechanisms in LN pathogenesis, dysregulation of RNASE1-mediated extracellular RNA clearance may contribute to renal inflammatory injury in SLE.

To identify biomarkers with both systemic and renal relevance, integrative analytical strategies combining multi-omics datasets have increasingly been applied. Cross-platform and cross-species integration can help prioritize molecules that are consistently dysregulated in disease-relevant contexts. In the present study, we integrated serum proteomic data from patients with SLE and transcriptomic data derived from the kidneys of SLE mouse models. Through this integrative approach, RNASE1 emerged as the only overlapping candidate molecule showing consistent dysregulation at both the circulating and renal levels.

Based on these findings, we further evaluated the clinical relevance of RNASE1 in SLE. Specifically, we assessed serum RNASE1 levels in healthy controls, patients with SLE, and patients with SLE complicated by LN, and analyzed their association with disease activity. We also investigated renal RNASE1 expression in LN tissues and analyzed its relationship with histopathological class and activity index. Finally, to improve clinical applicability, we developed an RNASE1-albumin-calcium (RAC) score and evaluated its performance in discriminating different levels of SLE disease activity.

## Materials and methods

### Patients enrollment and study approval

A total of 155 patients diagnosed with SLE, including 105 with LN, were consecutively recruited from The Second Affiliated Hospital of Anhui Medical University from April 2023 to February 2025. Among the LN patients, 29 were classified as class III ± V, 71 as class IV ± V, and 5 as class V. All patients fulfilled the revised classification criteria for SLE established by the American College of Rheumatology (ACR) (18). In addition, 81 age- and sex-matched people without autoimmune or inflammatory diseases were enrolled as healthy controls (HC). Among the patients with SLE, a subset of 10 newly diagnosed, treatment-naïve patients (including 5 with LN) was selected for serum proteomic analysis. None of these patients had received glucocorticoids, immunosuppressive agents, or biologic therapies prior to serum collection.

LN was defined by the presence of at least one of the following criteria: persistent proteinuria >0.5g/24h confirmed on at least two measurements performed within 3 months, or persistent hematuria (≥5 red blood cells per high-power field on microscopic urinalysis) detected in at least two consecutive urine samples and not attributable to other causes. Alternatively, LN was diagnosed based on renal biopsy findings consistent with lupus-related glomerular lesions, including membranous glomerulonephritis, proliferative glomerulonephritis, or other characteristic pathological features. Renal biopsy specimens were classified according to the International Society of Nephrology/Renal Pathology Society (ISN/RPS) classification criteria (19). Patients were excluded if they had other connective tissue diseases, active infections, malignancies, or were pregnant or breastfeeding at the time of enrollment. A detailed flow diagram illustrating patient enrollment, exclusion, and subgroup allocation is provided in **Supplementary Figure 1**.

Disease activity was assessed using the Systemic Lupus Erythematosus Disease Activity Index (SLEDAI), a validated composite index that evaluates global disease activity in patients with systemic lupus erythematosus based on clinical and laboratory parameters (20). Based on SLEDAI scores, patients were stratified into four groups: inactive disease (0–4), mild activity (5–9), moderate activity (10–14) and severe activity (≥15).

Demographic characteristics, clinical manifestations, and laboratory parameters, and pathological data were obtained from hospital medical records, and independently reviewed and verified by experienced physicians to ensure data accuracy. The clinical characteristics of all participants are summarized in **Table 1**. All participants provided written informed consent prior to enrollment. This protocol has been approved by the Ethics Committee of Anhui Medical University (NO. YX2021-107).

**Table 1:** Characteristics of SLE patients and healthy controls.

Variable	SLE group (n = 155) mean (95% confidence interval)	Healthy controls (n = 81) mean (95% confidence interval)
Females/males	124/31	60/21
Age, years	39 (37-42)	34 (32-36)
C3 (g/L)	0.84 (0.78-0.90)	
C4 (g/L)	0.19 (0.16-0.21)	
SLEDAI score	9.01 (8.07-9.96)	
ESR (mm/h)	17.99 (13.98-22.00)	
24h urinary protein (g/d)	1.523 (1.13-1.92)	
White Blood Cell (×10^9^/L)	6.55 (6.04-7.69)	6.48 (6.11-6.85)
Hemoglobin (g/L)	109.04 (105.48-112.60)	136.30 (132.51-140.09)
Platelet (×10^9^/L)	201.36 (186.96-215.75)	245.30 (230.66-259.55)
Albumin (g/L)	34.60 (33.37-35.83)	44.48 (43.79-45.18)
Serum Creatinine (umol/L)	80.19 (70.65-89.72)	54.77 (52.10-57.45)
Blood Urea Nitrogen (mmol/L)	7.73 (7.01-8.44)	4.77 (4.52-5.02)
RNASE1 (ng/mL)	38.012 (35.302-40.722)	25.074 (23.707-26.441)

### Serum samples preparation

All peripheral blood samples were collected into standard biochemical testing tubes from people recruited from the Second Affiliated Hospital of Anhui Medical University. All samples were processed within 2h of collection. Blood samples were centrifuged at 2000rpm for 5min at 4°C and the supernatant serum was carefully collected. Serum samples were aliquoted into sterile 1.5mL microcentrifuge tubes to avoid repeated freeze-thaw cycles and stored at -80°C until analysis. Prior to experimental use, samples were thawed at 4°C and gently mixed, and used immediately after thawing.

### Serum proteomic analysis

Serum samples were centrifuged to remove cellular debris, and the supernatant was collected for proteomic analysis. The 14 most abundant serum proteins were depleted using Pierce™ Top 14 Abundant Protein Depletion Spin Columns (Thermo Fisher Scientific). Protein concentrations were determined using a BCA assay.

Proteins were subsequently subjected to reduction, alkylation, and enzymatic digestion with trypsin following standard proteomic sample preparation procedures. The resulting peptides were separated using a reversed-phase nano-liquid chromatography system (EASY-nLC 1200, Thermo Fisher Scientific) and analyzed on an Orbitrap Exploris 480 mass spectrometer equipped with a nano-electrospray ion source.

Mass spectrometry data were acquired in data-dependent acquisition mode. The resulting spectra were processed using Spectronaut software (v17.0) with the Pulsar search engine and searched against the *Homo sapiens* UniProt reference proteome database. Carbamidomethylation of cysteine was set as a fixed modification, and oxidation of methionine and N-terminal acetylation were set as variable modifications. The false discovery rate was controlled at < 1% at the protein, peptide, and PSM levels. The resulting spectral library was subsequently used for subsequent DIA data analysis.

### Bioinformatics analysis

Quantitative proteomic data were processed using Spectronaut (v17.0), and protein intensities were normalized based on the default global normalization strategy. Proteins with missing values in more than 30% of samples were excluded from downstream analyses. Remaining missing values were imputed using a minimal value approach to approximate low-abundance proteins. Differentially expressed proteins (DEPs) between patients with SLE and healthy controls were identified using moderated t-tests. Proteins with an |log2 fold change (FC)| > 1 and a false discovery rate (FDR)-adjusted *P* value < 0.05 were considered significantly differentially expressed.

Functional enrichment analyses were performed to explore the biological relevance of the identified DEPs. Gene Ontology (GO) biological process (BP), cellular component (CC), and molecular function (MF) enrichment and gene set enrichment analysis (GSEA) were conducted using R software. Pathways with an adjusted *P* value < 0.05 were regarded as significantly enriched.

### Integrative cross-species analysis

Human serum proteomic data and mouse renal transcriptomic data were analyzed independently to identify differentially expressed proteins and genes using standard normalization and statistical procedures. Proteins identified in the human serum proteomic dataset were converted to their corresponding gene symbols. Human–mouse ortholog mapping was performed using the Ensembl database to identify conserved genes across species. Differentially expressed human genes derived from the proteomic dataset were then intersected with differentially expressed genes identified in the mouse kidney transcriptomic dataset. The overlapping candidates were visualized using a Venn diagram.

### Enzyme-linked immunosorbent assay

Serum levels of RNASE1 were quantified using a commercially available ELISA kit (catalog no. SEK13468; Sino Biological Inc., Beijing, China) according to the manufacturer’s instructions. All serum samples were assayed in duplicate, and the mean value was used for subsequent analyses. Briefly, absorbance was measured at 450nm using a microplate reader (Multiskan MK3, Thermo Scientific) immediately after completion of the assay. A standard curve was generated using serial dilutions of the provided RNASE1 standards, with RNASE1 concentration plotted on the x-axis and optical density values on the y-axis. Serum RNASE1 concentrations were calculated based on the corresponding standard curve. All samples from different study groups were measured under identical experimental conditions to minimize inter-assay variability.

### RAC score calculation

The RAC score was constructed to integrate serum RNASE1 levels with clinically relevant biochemical parameters for the assessment of SLE disease activity. To derive this composite score, an ordered logistic regression model was applied using disease activity as the dependent variable.

Candidate laboratory variables, including serum RNASE1 and routine biochemical parameters, were initially included as independent variables. Variables already incorporated into the SLEDAI scoring system were excluded to avoid collinearity. A stepwise selection procedure was applied to remove non-significant predictors, and the remaining variables were retained to construct the final regression-based model.

Based on the regression coefficients obtained from the ordered logistic model, the RAC score was calculated as follows: RAC score = −0.038 × RNASE1 (ng/mL) + 0.143 × Albumin (g/L) + 11.581 × Calcium (mmol/L).

Serum albumin and calcium were retained in the final model because they demonstrated independent associations with SLE disease activity and provided complementary clinical information regarding systemic inflammation, renal function, and metabolic status. The regression-derived coefficients were used as weights to construct the composite RAC score, ensuring that each component contributed proportionally to the overall score based on its statistical association with disease activity.

The RAC score was subsequently evaluated for its association with SLE disease activity and relevant clinical parameters. Receiver operating characteristic (ROC) curve analysis was performed to assess the ability of the RAC score to discriminate different levels of disease activity.

### Immunohistochemistry

Renal biopsy specimens from patients with LN were collected and fixed in formalin, embedded in paraffin, and sectioned at a thickness of 4μm. After deparaffinization and rehydration, antigen retrieval was performed. Endogenous peroxidase activity was blocked, followed by incubation with a blocking solution to reduce nonspecific binding. Sections were incubated overnight at 4°C with a primary antibody against RNASE1 (Abmart, Cat: PS08750, 1:100), and subsequently with a horseradish peroxidase–conjugated secondary antibody at 37°C for 30min. Immunoreactivity was visualized using diaminobenzidine (DAB) as the chromogen, with hematoxylin counterstaining.

RNASE1 expression was quantitatively assessed using ImageJ software. Representative high-power fields were randomly selected under identical imaging conditions. The RNASE1-positive area was calculated and expressed as a percentage of the total tissue area. The mean value from multiple fields was used for statistical analysis. All image analyses were performed in a blinded manner with respect to clinical and pathological data.

### Image analysis of RNASE1 immunohistochemistry

RNASE1 expression in kidney sections was quantified using ImageJ software. Briefly, stained sections were digitized using a high-resolution slide scanner or microscope camera. For each section, 5–10 non-overlapping fields (magnification ×200) were randomly selected for analysis.

Images were first converted to 8-bit grayscale and background-subtracted to reduce non-specific staining. The color deconvolution plugin was applied to isolate the DAB signal representing RNASE1 positivity. A consistent threshold was set across all images to distinguish positive staining from background. The area fraction (%Area) of RNASE1-positive staining was calculated as the ratio of positive pixels to total pixels in the selected field. The mean %Area from all analyzed fields per section was used for statistical comparisons. All image analyses were performed blinded to the sample identity to avoid bias.

### Statistical analysis

All statistical analyses were performed using SPSS (version 24.0), GraphPad Prism (version 9.0) and R (version 4.3.3). Data are presented as mean ± standard deviation (SD). Normality was assessed using the Shapiro-Wilk test. Comparisons between two groups were performed using the Student’s t-test for normally distributed data or the Mann-Whitney U test for non-normally distributed data. Comparisons among three or more groups were conducted using one-way analysis of variance (ANOVA) followed by Tukey’s *post hoc* test, or the Kruskal-Wallis test followed by Dunn’s multiple comparisons test, as appropriate. Categorical variables were compared using the chi-square test or Fisher’s exact test. Correlations between serum RNASE1 levels, RAC score, RNASE1-positive area, and clinical parameters (including SLEDAI, proteinuria, and complement C3) were evaluated using Spearman’s rank correlation coefficient. ROC curves were constructed to assess the diagnostic performance of RNASE1 and the RAC score in discriminating different levels of SLE activity. The area under the ROC curve (AUC) with 95% confidence interval (CI) was calculated. Optimal cutoff values were determined using the Youden index. *P* < 0.05 was considered statistically significant for all analyses.

## Results

### Integrated serum proteomic and renal transcriptomic analyses identify RNASE1 as a candidate biomarker

**Figure 1:**
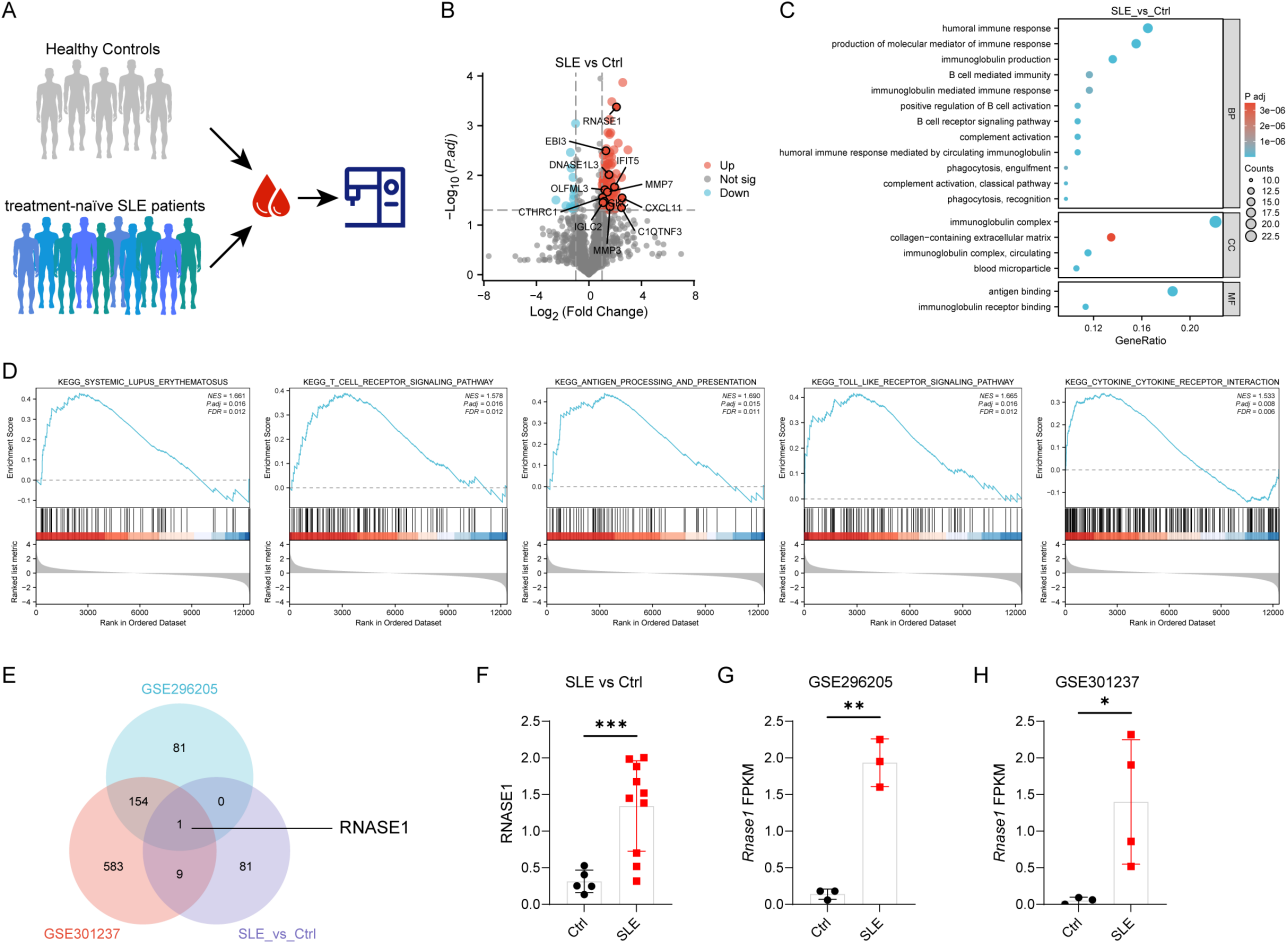
Integrated serum proteomic and renal transcriptomic analyses identify RNASE1 as a candidate biomarker in SLE. **(A)** Schematic workflow of the serum proteomic analysis. Serum samples were collected from an initial discovery cohort consisting of 10 newly diagnosed, treatment-naïve patients with SLE, including 5 patients with LN, and 5 healthy controls (HC), and subjected to mass spectrometry-based proteomic profiling. **(B)** Volcano plot showing differentially expressed serum proteins between SLE patients and healthy controls. Proteins with |log2 fold change (FC)| > 1 and *P* < 0.05 were considered significantly differentially expressed. **(C)** GO enrichment analysis of differentially expressed serum proteins, including biological process (BP), cellular component (CC), and molecular function (MF) categories. **(D)** GSEA of serum proteomic data, demonstrating significant enrichment of immune-related and SLE-associated pathways. Normalized enrichment scores (NES) and adjusted *P* values are indicated. **(E)** Venn diagram illustrating the overlap among differentially expressed serum proteins and renal DEGs identified from two independent lupus nephritis mouse transcriptomic datasets (GSE296205 and GSE301237). RNASE1 was identified as the only shared gene across all datasets. **(F, G)** Expression levels of RNASE1 in serum proteomic data **(F)** and renal transcriptomic datasets GSE296205 **(G)** and GSE301237 **(H)**. RNASE1 protein levels in serum and RNASE1 mRNA expression in renal tissues were compared between lupus and control groups. *P < 0.05, **P < 0.01, ***P < 0.001.

To identify potential differentially expressed serum proteins associated with SLE, serum samples from an initial discovery cohort consisting of 10 newly diagnosed, treatment-naïve patients with SLE (including 5 patients with LN) and 5 healthy controls were subjected to proteomic profiling (**Figure 1A**). Comparative analysis between the SLE and healthy control groups identified a set of differentially expressed proteins using the criteria of |log2 fold change (FC)| > 1 and *P* < 0.05 (**Figure 1B**).

GO enrichment analysis revealed that these differentially expressed proteins were predominantly involved in immune- and inflammation-related biological processes, including humoral immune response, immunoglobulin production, complement activation, B cell receptor signaling, and phagocytosis. Cellular component analysis showed significant enrichment in immunoglobulin complexes, while molecular function terms were mainly associated with antigen binding and immunoglobulin receptor binding (**Figure 1C**). To further explore the biological relevance of these serum proteomic alterations, GSEA was performed. The results demonstrated significant enrichment of pathways closely related to SLE pathogenesis, including cytokine-cytokine receptor interaction, antigen processing and presentation, Toll-like receptor signaling, T cell receptor signaling, and the systemic lupus erythematosus pathway itself (**Figure 1D**). Collectively, these findings indicate that the serum proteomic profile clearly distinguishes patients with SLE from healthy controls and is strongly associated with immune dysregulation characteristic of SLE.

To further identify candidate molecules linking systemic and renal involvement in SLE, renal transcriptomic datasets were obtained from two independent mouse models of LN, GSE296205 (17-week-old female MRL/lpr and C57BL/6 mice) and GSE301237 (21-week-old MRL/lpr mice and sex- and age-matched healthy controls). Differentially expressed genes (DEGs) were identified in each dataset using the criteria of |log2FC| > 1 and *P* < 0.05. The resulting renal DEGs from both datasets were mapped to their human orthologs and then intersected with the differentially expressed proteins identified in the serum proteomic analysis. This integrative approach revealed RNASE1 as the only gene consistently dysregulated across serum proteomics and both renal transcriptomic datasets (**Figure 1E**).

To further characterize its expression pattern, RNASE1 levels were examined in the serum proteomic data and in two independent renal transcriptomic datasets. In the serum proteomic analysis, RNASE1 levels were significantly elevated in patients with SLE, with a 4.29-fold increase compared with healthy controls (*P* < 0.001). Similarly, renal transcriptomic analysis showed significantly higher RNASE1 expression in SLE samples in both GSE296205 (13.81-fold increase vs. controls, *P* < 0.01) and GSE301237 (27.06-fold increase vs. controls, *P* < 0.05) (**Figures 1F–H**). The results showed that RNASE1 protein levels were significantly elevated in the serum of patients with SLE compared with healthy controls, and that RNASE1 mRNA expression was markedly increased in the kidneys of lupus-prone mice relative to control mice.

Together, these findings indicate that RNASE1 is consistently upregulated in both the circulation and renal tissue in SLE, supporting its potential role as a systemic biomarker linking immune dysregulation and renal involvement.

### Serum RNASE1 levels are significantly elevated in patients with SLE and correlate with disease activity and renal dysfunction

**Figure 2:**
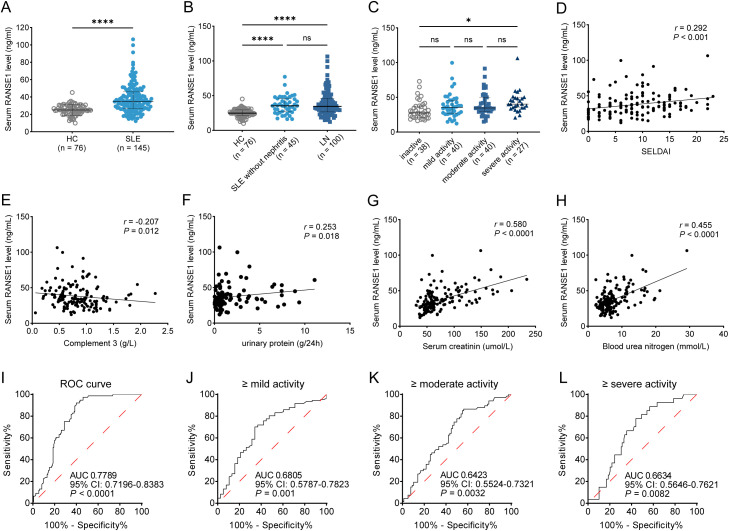
Serum RNASE1 levels are elevated in patients with SLE and correlate with disease activity and renal dysfunction. **(A)** Serum RNASE1 concentrations measured by ELISA in healthy controls (HC) and patients with SLE. **(B)** Comparison of serum RNASE1 levels among HC, patients with SLE without nephritis, and patients with LN. **(C)** Serum RNASE1 levels in patients with SLE stratified according to disease activity based on SLEDAI scores (inactive, mild, moderate, and severe). **(D, E)** Correlation analyses between serum RNASE1 levels and SLEDAI scores **(D)** and complement C3 levels **(E)** in patients with SLE. **(F–H)** Correlation analyses between serum RNASE1 levels and renal function parameters in patients with LN, including urinary protein excretion **(F)**, serum creatinine **(G)**, and blood urea nitrogen **(H)**. **(I)** ROC curve evaluating the performance of serum RNASE1 levels in discriminating patients with SLE from healthy controls. **(J–L)** ROC curve analyses assessing the discriminative ability of serum RNASE1 levels for identifying patients with ≥mild disease activity **(J)**, ≥moderate disease activity **(K)**, and ≥severe disease activity **(L)**. *P < 0.05, **P < 0.01, ***P < 0.001, ****P < 0.0001.

To validate the elevated expression of RNASE1 in the circulation of patients with SLE, serum samples were collected from an independent validation cohort comprising 145 patients with SLE, including 100 patients with LN, and 76 healthy controls. Serum RNASE1 concentrations were measured by ELISA. Compared with healthy controls, patients with SLE exhibited significantly higher serum RNASE1 levels, with a 1.52-fold increase relative to controls (*P* < 0.0001) (**Figure 2A**). However, no significant difference was observed between patients with SLE without nephritis and those with LN (**Figure 2B**).

Patients with SLE were further stratified according to disease activity. Serum RNASE1 levels were significantly higher in patients with severe disease activity compared with those with inactive disease, whereas patients with mild or moderate activity showed a trend toward increased RNASE1 levels that did not reach statistical significance (**Figure 2C**). To further explore the association between serum RNASE1 and disease activity, correlation analyses were performed. Serum RNASE1 levels were positively correlated with SLEDAI scores (*r* = 0.292, *P* < 0.001; **Figure 2D**) and negatively correlated with complement C3 levels (*r* = -0.207, *P* = 0.012; **Figure 2E**). These findings indicate a close association between circulating RNASE1 levels and disease activity in SLE.

In addition, the relationship between serum RNASE1 levels and renal function parameters was examined in patients with LN. Serum RNASE1 levels showed significant positive correlations with urinary protein excretion (*r* = 0.253, *P* = 0.018; **Figure 2F**), serum creatinine (*r* = 0.580, *P* < 0.001; **Figure 2G**), and blood urea nitrogen (*r* = 0.455, *P* < 0.001; **Figure 2H**), suggesting that elevated RNASE1 levels are associated with renal impairment in LN.

ROC curve analysis was performed to evaluate the diagnostic performance of serum RNASE1 levels in patients with SLE. Serum RNASE1 demonstrated good discriminative ability for distinguishing patients with SLE from healthy controls, with an AUC of 0.7789 (95% CI: 0.7196-0.8383, *P* < 0.0001; **Figure 2I**). We next assessed whether serum RNASE1 levels could discriminate different levels of disease activity among patients with SLE. ROC analysis showed that serum RNASE1 exhibited modest but statistically significant discriminative performance for identifying patients with ≥mild disease activity (AUC = 0.6805, 95% CI: 0.5787-0.7823, *P* = 0.001; **Figure 2J**), ≥moderate activity (AUC = 0.6423, 95% CI: 0.5524-0.7321, *P* = 0.0032; **Figure 2K**), and ≥severe activity (AUC = 0.6634, 95% CI: 0.5646-0.7621, *P* = 0.0082; **Figure 2L**) (**Table 2**). Together, these results indicate that serum RNASE1 has a relatively strong ability to distinguish patients with SLE from healthy individuals, while its performance in stratifying disease activity is moderate, suggesting that RNASE1 alone may be insufficient for precise activity discrimination but could contribute to activity assessment when combined with additional clinical parameters.

**Table 2:** Summary of ROC analysis results for RNASE1, RAC score, C3, C4, and proteinuria in stratifying disease activity levels in patients with SLE.

Comparison	AUC (95%CI)	Cut-off	Sensitivity (100%)	Specificity (100%)	PPV	NPV	Youden's index	P-value
RNASE1								
SLE vs HC	0.7789 (0.7196-0.8383)	31.84	60.00	92.11	93.55	54.69	0.5211	<0.0001
RNASE1								
≥mild activity	0.6805 (0.5787-0.7823)	31.59	70.09	65.79	85.23	43.86	0.3588	0.0010
≥moderate activity	0.6423 (0.5524-0.7321)	29.03	86.57	43.59	56.86	79.07	0.3016	0.0032
≥severe activity	0.6634 (0.5646-0.7621)	34.88	77.78	55.93	28.77	91.67	0.3371	0.0082
RAC score								
≥mild activity	0.7753 (0.6973-0.8533)	10.39	50.96	94.29	96.36	39.29	0.4525	<0.0001
≥moderate activity	0.7793 (0.7006-0.8580)	10.25	61.54	86.49	80.00	71.91	0.4803	<0.0001
≥severe activity	0.8086 (0.7222-0.8950)	10.54	84.00	68.42	36.84	95.12	0.5242	<0.0001
C3								
≥mild activity	0.7472 (0.6647-0.8297)	0.7950	58.88	81.58	90	41.33	0.4046	<0.0001
≥moderate activity	0.6682 (0.5784-0.7579)	0.8050	68.66	65.38	63.01	70.83	0.3404	0.0005
≥severe activity	0.8015 (0.7296-0.8733)	0.8050	96.30	60.17	35.62	98.61	0.5647	<0.0001
C4								
≥mild activity	0.7511 (0.6723-0.8299)	0.1550	60.75	84.21	91.55	43.24	0.4496	<0.0001
≥moderate activity	0.6677 (0.5771-0.7583)	0.1250	61.19	71.79	65.08	68.29	0.3298	0.0005
≥severe activity	0.7859 (0.7056-0.8663)	0.1450	88.89	61.02	34.29	96.00	0.4991	<0.0001
Proteinuria								
≥mild activity	0.7253 (0.6333-0.8174)	0.7680	49.00	93.10	96.08	34.62	0.4210	0.0002
≥moderate activity	0.7032 (0.6100-0.7965)	0.8925	55.74	85.29	77.27	68.24	0.4103	<0.0001
≥severe activity	0.6727 (0.5619-0.7835)	0.7680	66.67	67.65	35.29	88.46	0.3432	0.0059

### RAC score improves the assessment of disease activity in patients with SLE

**Figure 3:**
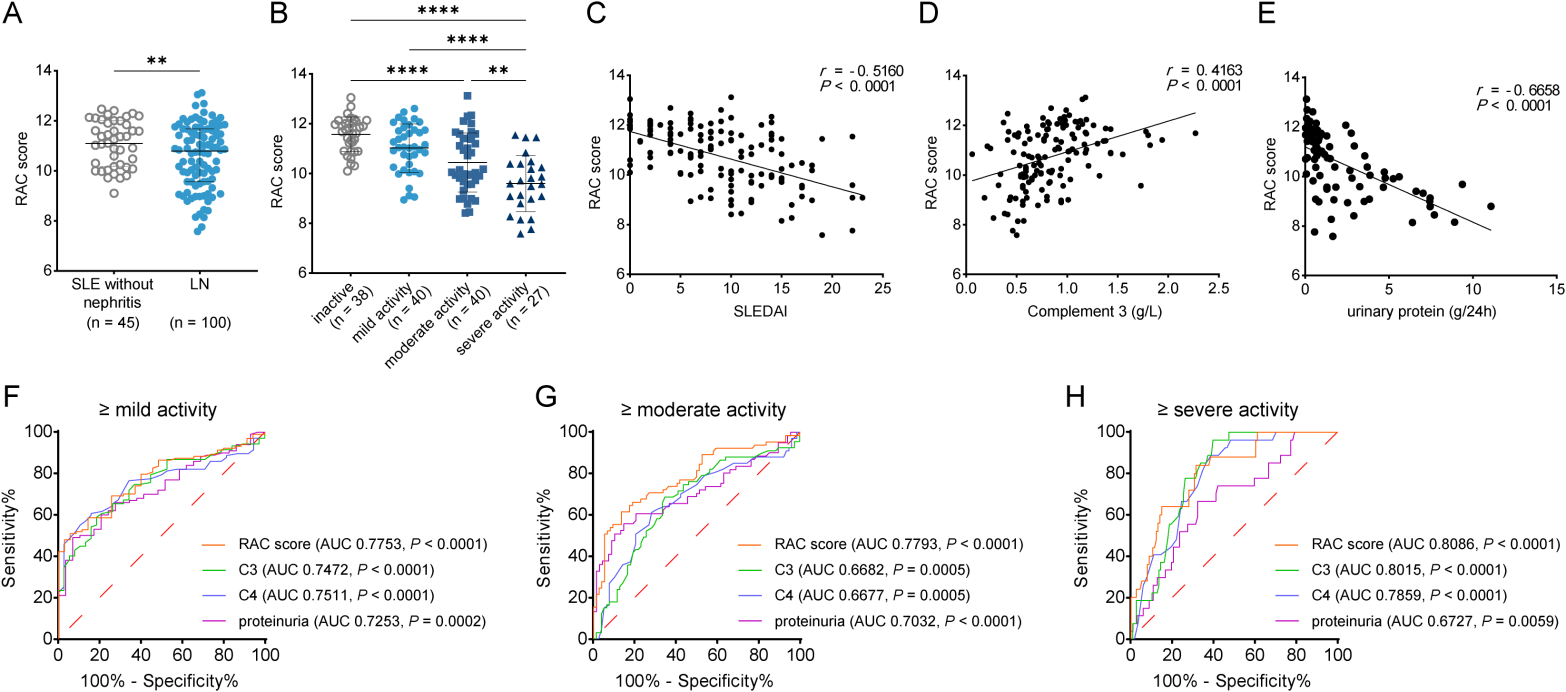
RAC score improves the assessment of disease activity in patients with SLE. **(A)** Comparison of RAC scores between patients with SLE without nephritis and patients with LN. **(B)** RAC scores in patients with SLE stratified according to disease activity based on SLEDAI scores (inactive, mild, moderate, and severe). **(C, D)** Correlation analyses between RAC scores and SLEDAI scores **(C)** and complement C3 levels **(D)** in patients with SLE. **(E)** Correlation analysis between RAC scores and urinary protein excretion in patients with SLE. **(F–H)** ROC curve analyses evaluating the performance of the RAC score for identifying patients with ≥mild disease activity **(F)**, ≥moderate disease activity **(G)**, and ≥severe disease activity **(H)**. **P < 0.01, ***P < 0.001, ****P < 0.0001.

To enhance the discrimination of disease activity in patients with SLE, an RAC score was constructed by integrating serum RNASE1 levels with routinely available laboratory parameters. Compared with patients with SLE without nephritis, patients with LN exhibited significantly lower RAC scores (*P* < 0.01; **Figure 3A**). When patients were stratified according to disease activity, RAC scores decreased progressively with increasing activity levels. Specifically, patients with severe disease activity exhibited RAC scores that were 0.83-fold of those in the inactive group (*P* < 0.0001) (**Figure 3B**). Correlation analyses further demonstrated a strong negative association between RAC scores and SLEDAI scores (*r* = -0.516, *P* < 0.0001; **Figure 3C**), and a strong positive correlation with complement C3 levels (*r* = 0.4163, *P* < 0.0001; **Figure 3D**). Notably, these correlations were substantially stronger than those observed with serum RNASE1 levels alone, indicating that the RAC score more accurately reflects overall disease activity. The association between RAC scores and renal involvement was also evaluated. RAC scores showed a significant negative correlation with urinary protein excretion (*r* = -0.6658, *P* < 0.0001; **Figure 3E**), further supporting the relevance of the RAC score to renal dysfunction in SLE. Again, the strength of this association exceeded that observed for serum RNASE1 alone.

ROC curve analyses were performed to assess the diagnostic performance of the RAC score for identifying different levels of disease activity. The RAC score demonstrated good discriminative ability for detecting ≥mild disease activity (AUC = 0.7753, 95% CI: 0.6973-0.8533, *P* < 0.0001; **Figure 3F**), ≥moderate activity (AUC = 0.7793, 95% CI: 0.7006-0.8580, *P* < 0.0001; **Figure 3G**), and ≥severe activity (AUC = 0.8086, 95% CI: 0.7222-0.8950, *P* < 0.0001; **Figure 3H**) (**Table 2**). For comparison, ROC analyses were also performed for conventional laboratory markers, including serum C3, C4, and proteinuria. Across all disease activity thresholds, the RAC score consistently yielded higher AUC values than these individual parameters (**Figures 3F, G**, **Table 2**). Collectively, these results indicate that the RAC score provides improved discriminative performance compared with serum RNASE1 alone and conventional laboratory markers for assessing disease activity across different severity thresholds in SLE.

### Renal RNASE1 expression is upregulated and correlates with pathological activity in lupus nephritis

**Figure 4:**
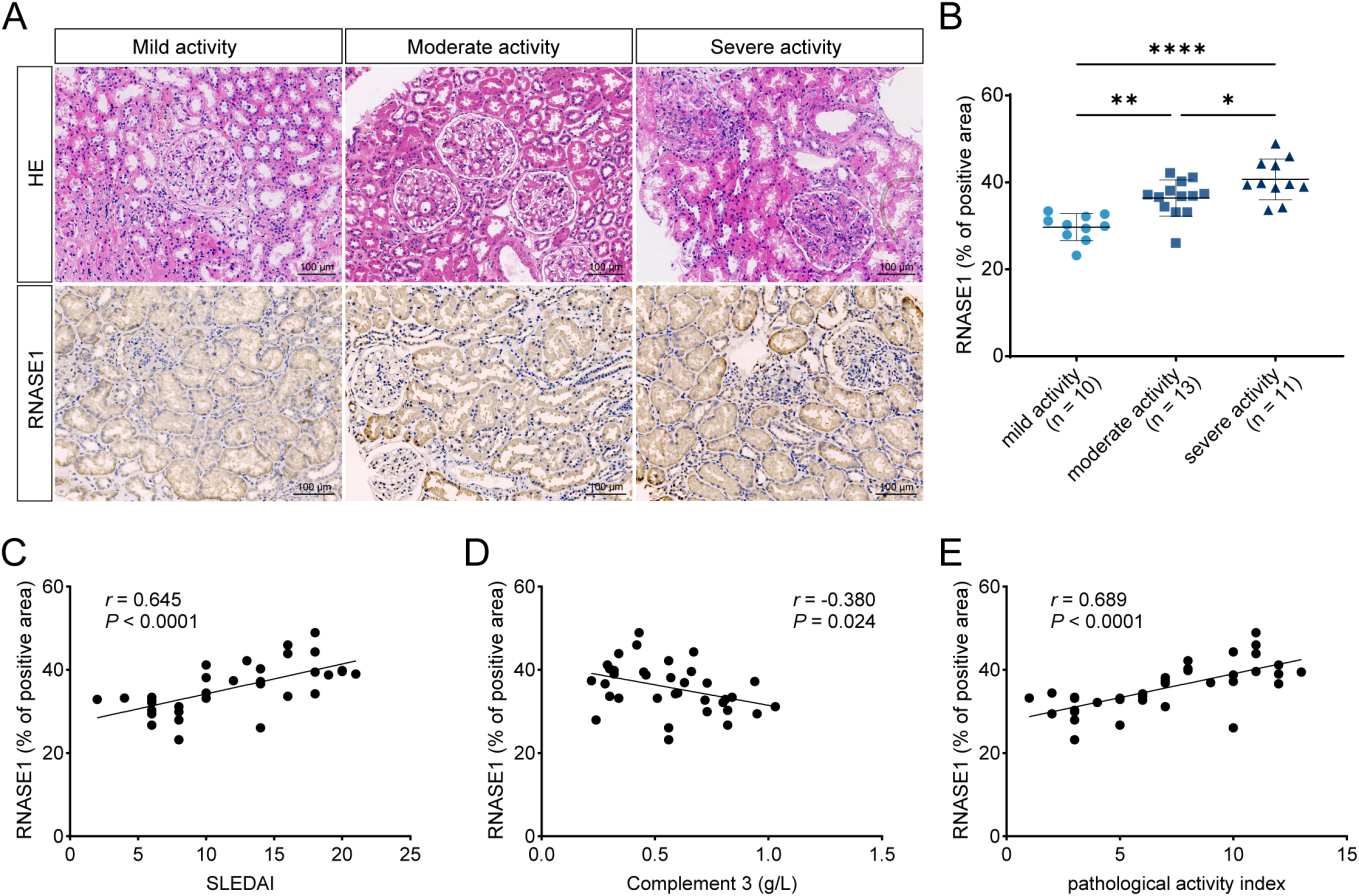
Renal RNASE1 expression is upregulated and correlates with pathological activity in lupus nephritis. **(A)** Representative hematoxylin and eosin (HE) and IHC staining of RNASE1 in renal biopsy specimens from patients with LN stratified by disease activity (mild, moderate, and severe). **(B)** Quantification of RNASE1-positive area in renal tissues from patients with LN according to disease activity, including mild activity (n = 10), moderate activity (n = 13), and severe activity (n = 11). **(C, D)** Correlation analyses between renal RNASE1 expression (positive staining area) and SLEDAI scores **(C)** or complement C3 levels **(D)** in patients with LN. **(E)** Correlation analysis between renal RNASE1 expression and the pathological activity index in patients with LN. *P < 0.05, **P < 0.01, ****P < 0.0001.

Given that RNASE1 was identified through integrative analyses of serum proteomics and renal transcriptomics, its expression in renal tissue was further examined in patients with LN. Immunohistochemical staining was performed on renal biopsy specimens from patients with LN, with adjacent non-tumorous renal tissues from patient with renal cell carcinoma used as control. RNASE1 expression was markedly increased in renal tissues from patients with LN compared with control tissue (**Supplementary Figure 2**). Patients with LN were subsequently stratified according to disease activity. Quantitative analysis of RNASE1 immunostaining revealed a significant increase in RNASE1-positive area with increasing disease activity (**Figures 4A, B**). Compared with the mild activity group, the moderate activity group showed a 1.23-fold increase (*P* < 0.01), while the severe activity group showed a 1.37-fold increase (*P* < 0.001). In addition, the severe group was 1.12-fold higher than the moderate group (*P* < 0.05) (**Figure 4B**). Correlation analyses further demonstrated that renal RNASE1 expression was positively correlated with SLEDAI scores (n = 36, *r* = 0.645, *P* < 0.0001; **Figure 4C**) and negatively correlated with complement C3 levels (n = 35, *r* = -0.380, *P* = 0.024; **Figure 4D**).

To evaluate the association between renal RNASE1 expression and histopathological severity, RNASE1-positive area was correlated with the pathological activity index. A strong correlation was observed between renal RNASE1 expression and pathological activity index (n = 36, *r* = 0.689, *P* < 0.0001; **Figure 4E**), indicating that increased RNASE1 expression in the kidney is closely associated with histological activity in LN.

Collectively, these findings demonstrate that RNASE1 is markedly upregulated in the kidneys of patients with LN and that its renal expression closely parallels both clinical disease activity and histopathological severity.

### Renal RNASE1 expression is associated with histopathological class in lupus nephritis

**Figure 5:**
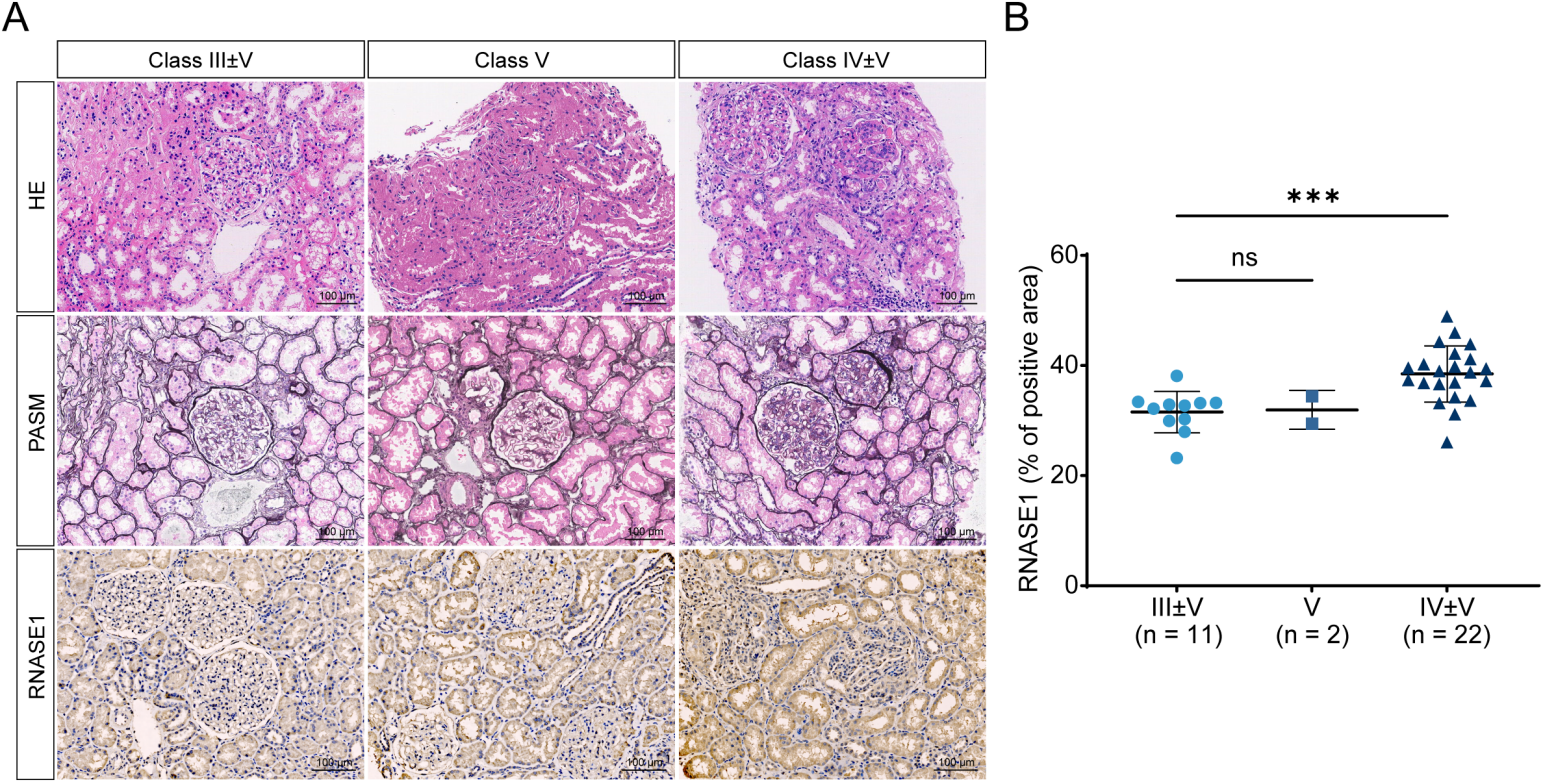
Renal RNASE1 expression is associated with histopathological class in lupus nephritis. **(A)** Representative HE, periodic acid-silver methenamine (PASM), and RNASE1 IHC staining of renal biopsy specimens from patients with different histopathological classes of LN, including class III ± V (n = 11), class IV ± V (n = 22), and class V (n = 2). **(B)** Quantification of RNASE1-positive area in renal tissues across different LN histopathological classes. RNASE1 expression was significantly higher in class IV ± V compared with class III ± V and class (V) *P < 0.05, **P < 0.01, ***P < 0.001, ***P < 0.0001.

To explore the association between renal RNASE1 expression and histopathological class in LN, patients with available immunohistochemical data were stratified according to the ISN/RPS classification. LN cases were grouped as class III with or without concomitant class V (class III ± V, n = 11), class IV with or without concomitant class V (class IV ± V, n = 22), and class V (n = 2). Renal RNASE1 expression levels were then compared among these pathological classes. Quantitative immunohistochemical analysis revealed that renal RNASE1 expression was significantly higher in class IV ± V LN compared with class III ± V LN, with class IV ± V showing a 1.22-fold increase relative to class III ± V (*P* < 0.001) (**Figures 5A, B**). These findings indicate that elevated renal RNASE1 expression is preferentially associated with proliferative forms of LN.

## Discussion

In the present study, we integrated serum proteomics with renal transcriptomic data to identify RNASE1 as a molecule consistently dysregulated in SLE and LN. We demonstrated that RNASE1 was significantly upregulated in the serum of patients with SLE and in the kidneys of patients with LN, and that its expression was closely associated with clinical disease activity, renal dysfunction, and histopathological severity. Moreover, by combining RNASE1 with routinely measured laboratory parameters, we constructed an RAC score that showed improved performance in assessing disease activity compared with serum RNASE1 alone. Together, these findings suggest that RNASE1 represents a systemic biomarker linking immune activation and renal involvement in SLE, and that the RAC score may provide a practical tool for noninvasive disease activity assessment.

The ribonuclease A (RNase A) superfamily comprises a group of secreted ribonucleases that share structural homology and are encoded by closely linked genes on chromosome 14 (21). In humans, this superfamily includes canonical members (RNases 1-8) that retain ribonucleolytic activity and noncanonical members (RNases 9-13) that lack one or more catalytic residues required for enzymatic function (21, 22). Canonical RNases, including RNASE1, have been implicated in extracellular RNA degradation (23), host defense (24), and immunomodulatory responses (14), whereas the functions of noncanonical members remain less well defined. RNASE1, originally characterized as the archetype of the RNase A family, is broadly expressed and contributes to the metabolism of extracellular RNA and maintenance of vascular homeostasis (11, 25). Previous studies have suggested that extracellular RNases participate in the degradation of circulating RNA released during tissue injury or inflammation, thereby limiting excessive immune activation (26, 27). In the context of SLE, a disease characterized by widespread immune dysregulation and chronic inflammation, increased RNASE1 expression may reflect a compensatory response to heightened inflammatory burden or endothelial stress. Notably, recent integrative analysis of the GSE50772 dataset, combining differential gene expression, weighted gene co-expression network analysis, and three machine learning algorithms, identified RNASE1, along with ARID2, CYSTM1, and DDIT3, as key immune-related biomarkers with high diagnostic potential in SLE (28). Despite these insights, the precise role of RNASE1 in systemic autoimmune diseases remains unclear. To our knowledge, this study is among the first to demonstrate consistent upregulation of RNASE1 in both the circulation and renal tissue of SLE patients, highlighting a potential link between RNASE1 expression and disease activity.

Importantly, serum RNASE1 levels were positively correlated with SLEDAI scores and negatively correlated with complement C3 levels, indicating that RNASE1 reflects global disease activity rather than isolated clinical manifestations. Although serum RNASE1 alone showed only moderate performance in discriminating different levels of disease activity by ROC analysis, this observation is consistent with the well-recognized limitation that single biomarkers rarely capture the complexity of SLE activity. Nevertheless, the consistent association between RNASE1 levels and multiple clinical parameters suggests that RNASE1 carries biologically meaningful information related to disease activity.

To improve clinical applicability, we integrated RNASE1 with serum albumin and calcium levels to construct the RAC score. Albumin and calcium are routinely measured laboratory parameters that reflect systemic inflammation, nutritional status, and renal involvement, all of which are frequently altered in active SLE (29–31). The RAC score demonstrated substantially stronger correlations with SLEDAI, complement C3, and urinary protein excretion than RNASE1 alone. In addition, ROC analyses showed that the RAC score achieved good discriminative performance across different activity thresholds, with AUC values exceeding those of serum RNASE1 alone. These findings indicate that combining RNASE1 with complementary clinical indicators enhances its ability to capture disease activity and supports the utility of composite scores in complex autoimmune diseases such as SLE.

Renal involvement represents a major determinant of prognosis in SLE (5), and accurate assessment of LN activity remains a clinical challenge. In this study, serum RNASE1 levels did not significantly differ between patients with SLE with and without lupus nephritis in our cohort. This observation suggests that circulating RNASE1 may reflect systemic immune activation rather than kidney-specific injury. Given that RNASE1 is primarily produced by vascular endothelial cells and is involved in extracellular RNA-mediated inflammatory signaling, its elevation may represent a broader inflammatory response in SLE. Notably, when integrated with albumin and calcium into the RAC score, the composite index demonstrated improved discrimination between patients with and without lupus nephritis, indicating that multi-parameter models may better capture the complex pathophysiological features of renal involvement. Additionally, renal RNASE1 expression was markedly increased in LN tissues compared with control renal tissues and showed a strong positive correlation with pathological activity index. Furthermore, RNASE1 expression was significantly higher in class IV ± V LN compared with class III ± V LN. These results suggest that RNASE1 expression is preferentially associated with proliferative and histologically active forms of LN, which are known to carry a higher risk of progression and worse renal outcomes (32, 33).

Notably, the parallel upregulation of RNASE1 in both serum and renal tissue highlights its potential role as a systemic-renal link in SLE. This finding is particularly relevant given the need for noninvasive biomarkers that can reflect renal pathology without repeated kidney biopsies. Although renal biopsy remains the gold standard for LN classification and activity assessment, circulating biomarkers such as RNASE1 and composite indices like the RAC score may complement histopathological evaluation and aid in disease monitoring.

A notable strength of this study is that the discovery cohort consisted exclusively of newly diagnosed, treatment-naïve patients with SLE, minimizing the confounding effects of prior therapy and disease duration on serum proteomic profiles. Given the difficulty of recruiting treatment-naïve patients, particularly those with lupus nephritis, the inclusion criteria were broadened in the validation cohort to better reflect a real-world clinical population. Despite this heterogeneity, RNASE1 remained consistently associated with disease activity and renal involvement, supporting the robustness of our findings across different disease stages and treatment conditions.

Several limitations of this study should be acknowledged. First, the serum proteomic discovery cohort was relatively small, which may have limited the number of differentially expressed proteins detected. Consequently, only RNASE1 overlapped with the murine renal transcriptomic dataset. Second, this was a single-center study with a relatively limited sample size, which may restrict the generalizability of the findings. Third, the cross-sectional design precludes assessment of longitudinal changes in RNASE1 and RAC scores in response to treatment or during disease progression, treatment, or remission, and the potential influence of ongoing therapy on RNASE1 expression cannot be fully excluded. Finally, although our results demonstrate strong associations between RNASE1 expression and disease activity, the mechanistic role of RNASE1 in SLE and LN pathogenesis, such as the precise cellular and structural localization of RNASE1 in the kidney and its potential interactions with infiltrating immune cells or immune complex deposition, were not directly addressed and warrants further investigation using experimental models. Future studies should therefore include larger, multi-center cohorts, longitudinal sampling, and experimental models to clarify the dynamic behavior and functional contribution of RNASE1 in immune regulation and renal inflammation.

In conclusion, this study identifies RNASE1 as a molecule consistently associated with systemic and renal disease activity in SLE. The RNASE1-based RAC score improves the assessment of disease activity and represents a promising, noninvasive tool for evaluating disease severity, particularly in patients with lupus nephritis.

## Data Availability

The original contributions presented in the study are included in the article/**Supplementary Material**. Further inquiries can be directed to the corresponding authors.
