# Supplementary material for: Serum RNASE1 as a biomarker for disease activity and lupus nephritis in systemic lupus erythematosus
Source: Front Immunol. 2026 Apr 1;17:1790455. doi: 10.3389/fimmu.2026.1790455 (PMC13079621; doi:10.3389/fimmu.2026.1790455)
Supplement: Supplementary file 1 [file Supplementaryfile1.docx]

Figure S1


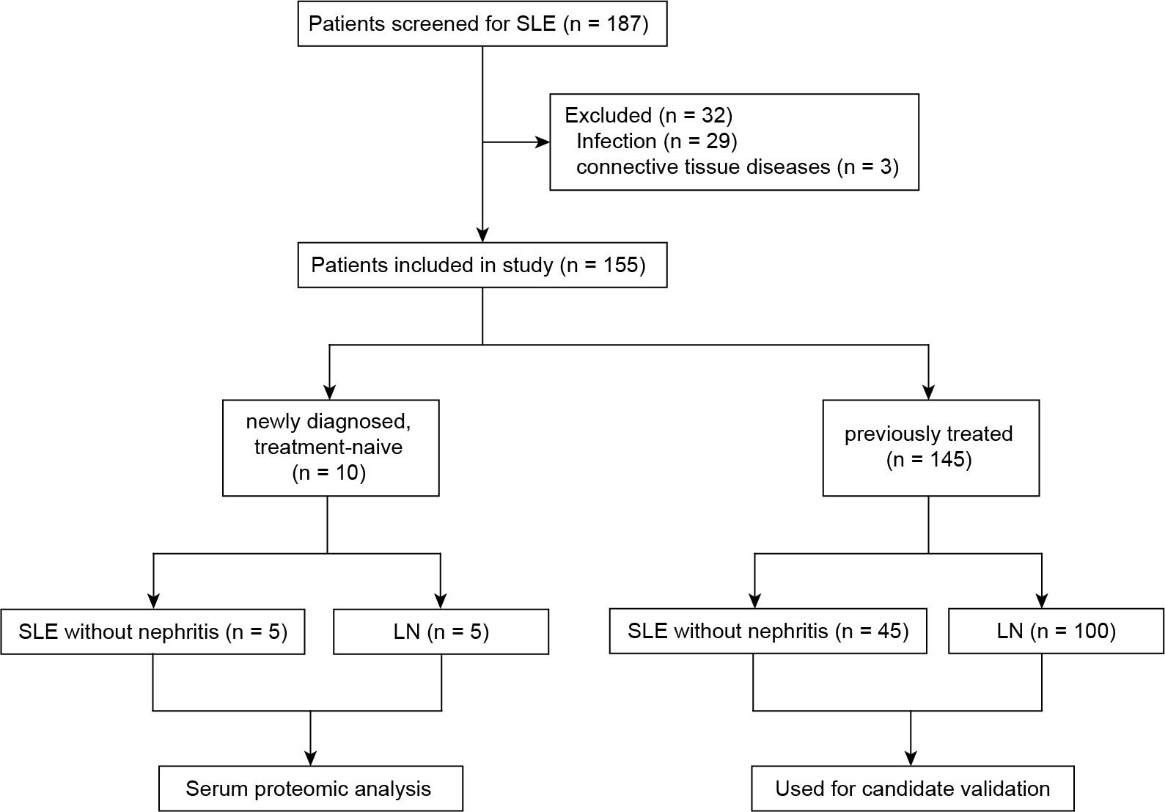


**Figure S1. Flow diagram of patient enrollment and subgroup allocation.**

A total of 187 patients with SLE were initially screened. Among them, 29 patients were excluded due to active infections and 3 patients were excluded due to other connective tissue diseases, leaving 155 patients included in the study. These patients were further divided into treatment‑naïve, newly diagnosed patients (n = 10) and previously treated patients (n = 145). The treatment‑naïve group was split into LN (n = 5) and SLE without nephritis (n = 5) subgroups and used for serum proteomics discovery. The treated group was split into LN (n = 100) and SLE without nephritis (n = 45) subgroups and used for candidate validation.

Figure S2


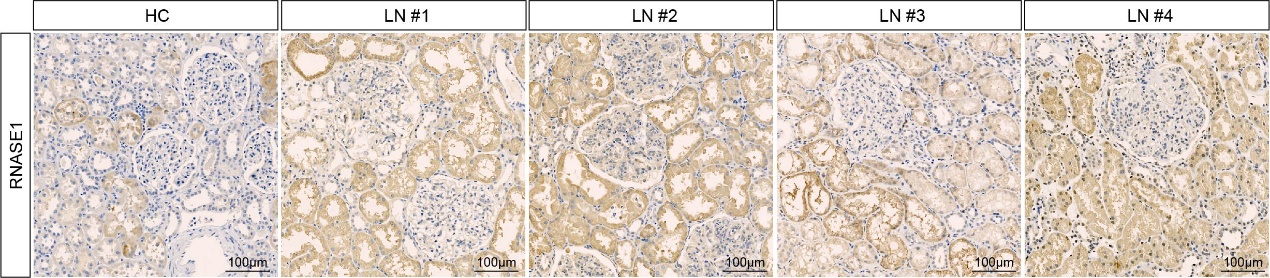


**Figure S1. Renal RNASE1 expression is increased in lupus nephritis compared with control renal tissue**

Representative IHC staining of RNASE1 in renal biopsy specimens from patients with LN and adjacent non-tumorous renal tissues from patients with renal cell carcinoma used as HC. RNASE1 expression is markedly increased in renal tissues from patients with LN compared with control tissues.
